# IL-10 -1082 A/G polymorphism is related with the risk and clinical characteristics of acute kidney injury: a case-control study

**DOI:** 10.1186/s12882-021-02410-1

**Published:** 2021-06-05

**Authors:** Haiyan Mu, Qingqing Zheng, Lihai Hao

**Affiliations:** 1 Health Management center, Weifang People’s Hospital, 151 Guangwen Street, Kuiwen District, Shandong Province 261041 Weifang, China; 2 Hemodialysis Center, Weifang People’s Hospital, 151 Guangwen Street, Kuiwen District, Shandong Province 261041 Weifang, China

**Keywords:** IL-10, acute kidney injury, -1082 A/G, polymorphism

## Abstract

**Background:**

Interleukin-10 (IL-10), a kind of anti-inflammation cytokine, has a key role in the development of acute kidney injury (AKI). Recently, several studies addressed the link between the risk of AKI and the IL-10 -1082 A/G polymorphism with conflicting findings.

**Methods:**

To identify the effects of the IL-10 -1082 A/G polymorphism on the risk of AKI, we designed this case-control study. This study recruited 320 AKI patients and 408 ICU patients without AKI. The association between the AKI risk and this polymorphism was analyzed using the logistic regression analysis adjusted for confounding factors.

**Results:**

The IL-10 -1082 A/G polymorphism enhanced the risk of AKI. After stratified analysis, this polymorphism increased the risk of AKI among the males, smokers, those aged exceeding 60 years old, and overweight individuals (BMI ≥ 25). Moreover, -1082 A/G polymorphism was remarkably related with APACHE II score and eGFR.

**Conclusions:**

Collectively, the IL-10 -1082 A/G polymorphism is linked with an elevated risk of AKI. Further studies in China need be performed to verify these results.

## Introduction

Acute kidney injury (AKI) is a group of complex syndromes characterized by serum creatinine increasing apace or urine produced decrease [1]. AKI occurs in roughly 10–15 % of hospitalized patients, and its incidence in Intensive care unit (ICU)was more than 50 % of patients [1]. The AKI-related mortality rates were about13.8 % in children and 23.9 % in adults among hospitalized patients [2]. The prevalence of AKI is gradually increasing, and early detection and diagnosis of AKI is associated with decreased mortality [3]. The risk elements of AKI are multiple such as older age, male sex, genetic factor, and comorbidities such as diabetes, proteinuria, heart failure, sepsis and hypoalbuminaemia [4]. GWAS studies showed the association between novel loci in certain genes and susceptibility to AKI [5–7]. Additionally, previous studies revealed that the pathogenesis of AKI was extremely complex and extensive, which was involved in almost all aspects of the cytokines, innate immunity, humoral and cellular immunity [8]. Thus, the variants of some cytokines may correlate with the susceptibility to AKI.

Interleukin (IL)-10 is a pivotal anti-inflammatory cytokine, which is primarily produced by blood cells and organ cells. IL-10 has a key role in restricting excessive inflammatory responses, which could upregulate innate immunity and promote tissue repairing mechanisms to keep tissue homeostasis during inflammation and infection [9]. Previous studies indicated that cytokines including IL-10, IL-6, and TNF-α released by relation-immune cells when the renal ischemia/reperfusion occurred [10]. Li et al. showed that IL-10 levels were markedly elevated in the AKI patients compared with the non-AKI individuals [11]. In addition, they indicated that IL-10 was positive related with the C-reactive protein (CRP) levels and urinary occult blood [11]. IL-10 exerted protective effects on renal ischemia-reperfusion injury by suppressing the secretion of pro-inflammatory cytokines and the expression of pro-apoptosis factors [12]. Furthermore, Wang et al. indicated that IL-10 derived from dendritic cells protected against cisplatin-induced nephrotoxicity [13]. Animal studies revealed that IL-10 could improve renal function and mitigate systemic inflammation following ischemic AKI in mice [14]. For its application prospects, Wang et al. found that extracellular vesicle-encapsulated IL-10 could be regarded as novel nanotherapeutics against AKI [15].

IL-10 gene is shown to be located on chromosome 1q32.1. Rs1800896 polymorphism is an important locus in the promoter region of IL-10 gene [16]. Recently, several studies interpreted the link between the IL-10 -1082 A/G polymorphism and risk of kidney diseases [17–21]. However, no studies paid attention to the relationship between this polymorphism and the risk of AKI. Therefore, we performed this retrospective study to explore the potential relationship between the IL-10 -1082 A/G polymorphism and the risk of AKI in Chinese Han individuals.

## Patients and methods

### Subjects

This study recruited 320 patients with AKI and 408 controls. AKI was diagnosed according to selection criteria by the KDIGO guideline [4, 22]. We collected clinicopathological characteristics including sex, age, smoking information, drinking information, body mass index (BMI), creatinine (mg/dl), APACHE II score, estimated glomerular filtration rate (eGFR), AKI stage, renal replacement therapy (RRT), and causes of AKI. eGFR was evaluated by using the Cockcroft–Gault formula. The individuals in control group were ICU patients without AKI from this hospital during the same period. All participating subjects undersigned informed consent. The Ethics Committee of this hospital approved the conduction of this retrospective study. Then, this study was in line with the *Helsinki declaration*.

### Genotyping

Blood samples of all participants were to extract DNA (Tiangen Biotech, Beijing, China). Relevant polymorphisms were genotyped using the PCR-RFLP method. In order to assess the genotyping error, we selected 10 % samples to re-genotype.

### Statistical analysis

categorical variables were calculated by the Chi-square (χ^2^) test; continuous variables were addressed by the t test. For controls, a good-fitness χ^2^ test was performed to calculate the *P-*value of the HWE test. Logistic regression analysis with adjustment of age, sex, BMI, smoking, and alcohol status was utilized to calculate odds ratios (ORs) and 95 % confidence intervals (95 % CIs). A *P-*value < 0.05 was showed as remarkable difference. All statistical analysis was calculated by SPSS 21.0.

## Results

### Clinicopathological characteristics of all investigated individuals

320 AKI cases and 408 matched controls from ICU were investigated, and they aged 52.17 ± 8.19 and 52.64 ± 7.98 years old, respectively. As for age, sex, smoking, alcohol and BMI, we did not observe evident differences between two groups (*P* > 0.05). The creatinine levels in AKI patients were markedly higher than in controls (*P* < 0.05). Other specific clinical characteristics of AKI are listed in Table 1.

**Table 1:** Clinicopathological characteristics of AKI patients and controls

Characteristics	AKI (n = 320)	Control (n = 408)	*P*
Mean age (SD)	52.17 ± 8.19	52.64 ± 7.98	0.432
Sex			0.784
Male	163(50.9 %)	212(52.0 %)	
Female	157(49.1 %)	196(48.0 %)	
Smoking			0.544
Yes	134(42.9 %)	180(44.1 %)	
No	186(58.1 %)	228(56.9 %)	
Alcohol			0.880
Yes	148(46.2 %)	191(46.8 %)	
No	172(53.8 %)	217(53.2 %)	
Mean BMI (SD)	25.85 ± 2.97	25.72 ± 2.93	0.576
Mean creatinine (SD), mg/dl	2.86 ± 1.18	0.88 ± 0.30	**0.000**
Mean APACHE II score (SD)	21.07 ± 5.31		
RRT, (n, %)	82(25.6 %)		
Baseline eGFR < 60 ml/(min/1.73m^2^)			
Yes	210(65.6 %)		
No	110(34.4 %)		
AKI stage (n, %)			
Stage 1	115(35.9 %)		
Stage 2	134(41.9 %)		
Stage 3	71(22.2 %)		
Causes of AKI, (n, %)			
Ischemic	100(31.3 %)		
Sepsis	65(20.3 %)		
Nephrotoxic	64(20.0 %)		
Obstructive	75(23.4 %)		
Others	16(5.0 %)		

SD, Standard Deviation; BMI, Body Mass Index; APACHE, Acute Physiology and Chronic Health Evaluation; RRT, renal replacement therapy; eGFR, estimated glomerular filtration rate; AKI, acute kidney injury. Bold values are statistically significant (*P* < 0.05)

### Relationship between the AKI risk and the IL-10 -1082 A/G polymorphism

This polymorphism distribution in controls conformed to the HWE test (*P*_*HWE*_ = 0. 970). Table 2 summarizes the allele and genotype distributions of this polymorphism. After logistic regression analysis, this polymorphism presented an association with an increased risk of AKI by adjusting age, sex, BMI, smoking and drinking (GG versus AA, adjusted OR, 1.84; 95 %CI, 1.07–3.15; *P* = 0.027; AG + GG versus AA, adjusted OR, 1.36; 95 %CI, 1.02–1.83; *P* = 0.040). Moreover, G allele carriers elevated the risk of AKI (G versus A, OR, 1.32; 95 % CI, 1.05–1.66; *P* = 0.017).

**Table 2:** Genotype frequencies of the IL-10 -1082 A/G polymorphism among cases and controls

Models	Genotype	Case (n, %)	Control (n, %)	OR (95 % CI)	*P*-value	*OR (95 % CI)	*P-value
	AA	148(46.3 %)	220(53.9 %)	1.00(reference)	-	1.00(reference)	-
Heterozygote	AG	137(42.8 %)	159(39.0 %)	1.28(0.94–1.75)	0.117	1.28(0.94–1.74)	0.120
Homozygote	GG	35(10.9 %)	29(7.1 %)	**1.79(1.05–3.06)**	**0.032**	**1.84(1.07–3.15)**	**0.027**
Dominant	AA	148 (46.3 %)	220(53.9 %)	1.00(reference)	-	1.00(reference)	-
	AG + GG	172(53.7 %)	188(46.1 %)	**1.36(1.01–1.82)**	**0.040**	**1.36(1.02–1.83)**	**0.040**
Recessive	AG + AA	285(89.1 %)	379(92.9 %)	1.00(reference)	-	1.00(reference)	-
	GG	35(10.9 %)	29(7.1 %)	1.61(0.96–2.69)	0.072	1.64(0.98–2.76)	0.062
Allele	A	433(67.7 %)	599(73.4 %)	1.00(reference)	-	1.00(reference)	-
	G	207(32.3 %)	217(26.6 %)	**1.32(1.05–1.66)**	**0.017**	**-**	**-**

*Adjusting age, sex, BMI, smoking and alcohol. Bold values are statistically significant (*P* < 0.05)

OR, Odds Ratios; 95 %CI, 95 % Confidence Intervals

Next, we interpreted the effects of this locus on the risk of AKI among the subgroups stratified by age, sexy, smoking, alcohol, and BMI in Table 3. We found that the − 1082 A/G polymorphism in IL-10 gene was related with an elevated risk of AKI among males, smokers, individuals over 60 years old, and overweight individuals (BMI ≥ 25).

**Table 3:** Stratified analyses regarding the association between the IL-10 -1082 A/G polymorphism and risk of AKI

Variables	Model 1 OR (95 % CI); *P*-value	Model 2 OR (95 % CI); *P*-value	Model 3 OR (95 % CI); *P*-value	Model 4 OR (95 % CI); *P*-value
Sex				
Male	1.30(0.84-2.00); 0.233	**2.99(1.32–6.76); 0.007**	**2.67(1.20–5.90); 0.013**	1.49(0.99–2.24); 0.059
Female	1.25(0.80–1.95); 0.322	1.17(0.57–2.44); 0.667	1.06(0.52–2.13); 0.877	1.24(0.81–1.88); 0.323
Age (years)				
< 60	1.34(0.96–1.88); 0.089	1.47(0.78–2.77); 0.233	1.29(0.70–2.38); 0.421	1.36(0.98–1.88); 0.063
≥ 60 BMI < 25 ≥ 25	1.02(0.48–2.18); 0.956 1.05(0.65–1.70); 0.843 1.48(0.99–2.21); 0.058	**2.94(1.03–8.42); 0.040** 1.97(0.90–4.33); 0.086 1.62(0.78–3.38); 0.193	**2.91(1.08–7.87); 0.030** 1.93(0.91–4.10);0.083 1.37(0.67–2.78);0.388	1.38(0.69–2.75); 0.366 1.19(0.76–1.88); 0.450 **1.50(1.02–2.20); 0.039**
Alcohol				
Yes	1.32(0.84–2.08); 0.234	1.98(0.92–4.23); 0.076	1.75(0.84–3.65); 0.131	1.43(0.93–2.20); 0.105
No	1.25(0.82–1.90); 0.304	1.64(0.77–3.47); 0.197	1.48(0.72–3.05); 0.290	1.30(0.87–1.95); 0.196
Smoking				
Yes	**1.92(1.19–3.11); 0.008**	**2.14(1.00-4.62); 0.048**	1.60(0.77–3.33); 0.207	**1.97(1.25–3.09); 0.003**
No	0.95(0.64–1.43); 0.822	1.60(0.76–3.39); 0.214	1.64(0.79–3.39); 0.180	1.04(0.70–1.53); 0.859

Model 1, AG vs. AA; Model 2, GG vs. AA; Model 3, GG vs. AA + AG; Model 4, GG + AG vs. AA; Bold values are statistically significant (*P* < 0.05)

OR, Odds Ratios; 95 %CI, 95 % Confidence Intervals; BMI, Body Mass Index; AKI, acute kidney injury

### Association with the IL-10 -1082 A/G polymorphism and relevant clinicopathological features of AKI

Last but not least, we revealed the linkage between this variant and the clinical features of AKI in Table 4. Data indicated that this polymorphism was related to the APACHE II score and eGFR among AKI patients. In addition, the − 1082 A/G polymorphism in IL-10 gene was correlated with the risk of ischemic and septic AKI when compared with corresponding control groups.

**Table 4:** The associations between the IL-10 -1082 A/G polymorphism and clinical characteristics of AKI

Characteristics			
	AG vs. AA	GG vs. AA	AG + GG vs. AA
	OR (95 %CI); *P*-value	OR (95 %CI); *P*-value	OR (95 %CI); *P*-value
APACHE II score			
≥22/<22	1.32(0.83–2.12); 0.241	**2.20(1.04–4.67); 0.037**	1.47(0.94–2.29); 0.090
RRT			
Yes/No	1.00(0.61–1.62); 0.986	1.23(0.58–2.62); 0.590	1.04(0.66–1.65); 0.865
AKI Stage			
Stage 2/Stage 1	1.05(0.62–1.77); 0.867	1.35(0.56–3.26); 0.500	1.10(0.67–1.81); 0.716
AKI Stage			
Stage 3/Stage 1	1.00(0.53–1.88); 0.992	1.72(0.65–4.57); 0.275	1.12(0.62–2.02); 0.714
Baseline eGFR < 60 ml/min/1.73m^2^			
Yes/No	**1.97(1.19–3.25); 0.008**	1.19(0.56–2.54); 0.659	**1.76(1.11–2.80); 0.017**
Cause of AKI			
Ischemic/Non-Ischemic	**2.36(1.40–3.97); 0.001**	**2.42(1.11–5.28); 0.024**	**2.37(1.44–3.89); 0.001**
Cause of AKI			
Sepsis/Non-sepsis	**2.48(1.33–4.63); 0.004**	**3.77(1.60–8.86); 0.001**	**2.72(1.50–4.93); 0.001**

OR, Odds Ratios; 95 %CI, 95 % Confidence Intervals; APACHE, Acute Physiology and Chronic Health Evaluation; RRT, renal replacement therapy; eGFR, estimated glomerular filtration rate; AKI, acute kidney injury. Bold values are statistically significant (*P* < 0.05)

## Discussion

This study discovered that the − 1082 A/G polymorphism in IL-10 gene enhanced the risk of AKI in Chinese Han people. Subgroup analysis indicated that this polymorphism elevated the risk of AKI among the males, smokers, those aged ≥ 60 years old, and overweight individuals (BMI ≥ 25).

Previous studies indicated that inflammatory reaction played a crucial role in the pathogenesis of AKI [23]. Both Th1 and Th2 derived cytokines elevated immediately after cardiac surgery and were related with AKI and its mortality [24]. Among these cytokines, IL-10 is a recognized anti-inflammatory cytokine, which helps to promote tissue homeostasis and maintain tissue integrity due to its characteristics as anti-apoptotic, anti-inflammatory and tissue regeneration properties [9]. IL-10 inhibits inflammatory and cytotoxic pathways implicated in AKI. Persistent-AKI patients showed significant decline of IL-10 levels [25].

The link between the risk of kidney diseases and the IL-10 -1082 A/G polymorphism has been explored before. Jaber et al. indicated that G allele carriers who produced higher levels of IL-10 was linked with a lower risk of mortality among acute renal failure patients [20] which was in line with the findings by Dalboni et al. [18]. A subsequent Taiwanese study suggested that this polymorphism was related to an elevated risk of contrast-induced nephropathy after percutaneous coronary intervention [17]. However, another Asian study from Japan uncovered that this polymorphism did not connect with the risk of chronic kidney disease and AKI following liver transplantation [21]. Dalboni et al. found that the − 1082 A/G polymorphism in IL-10 gene did not associate with the risk of AKI in Brazilians, although they observed TNF-α plus IL-10 low producer phenotype predicted AKI and death [18]. For septic AKI, an Egyptian study found that this polymorphism was related with a higher risk of AKI in severe sepsis patients [19]. In this study, we revealed that the − 1082 A/G polymorphism in IL-10 gene intensified the risk of AKI in a Chinese Han population. To our knowledge, this is the first study to reveal a link between the risk of AKI and the IL-10 -1082 A/G polymorphism in Chinese Han nationality from the mainland.

Obviously, abovementioned studies yielded inconsistent findings regarding the association between this polymorphism (-1082 A/G) and the risk of kidney diseases. The following aspects may be potential reasons. One, the dominant reason may due to disease heterogeneity. The Brazilian study by Dalboni et al. indicated that this locus was not related with the risk of AKI in the ICU patients [18]. However, the AKI patients in this study were because of ischemic, nephrotoxic, obstructive, and infectious factors. Two, different ethnicities were also important factors. For example, Kamei et al. showed that this polymorphism did not correlate with the risk of AKI in a Japanese population [21] while we observed that the − 1082 A/G polymorphism enhanced the risk of AKI in Chinese Han individuals. Three, the sample sizes may also account for it. Limited sample sizes may produce false positive or false negative results, which may exert effects on the final conclusions.

Subgroup analysis showed the IL-10 -1082 A/G polymorphism strengthened the risk of AKI among males, smokers, older individuals (age ≥ 60 years), and those overweight patients (BMI ≥ 25). Last, we found that this polymorphism was markedly related with APACHE II score, and eGFR in AKI individuals. Furthermore, this study indicated that this polymorphism was connected to the risk of ischemic and septic AKI.

Several limitations were shown in this study. First, the sample size of this retrospective study was not large enough; we only enrolled 320 AKI patients and 408 controls. Further large-scale studies are necessary to verify our results. Second, the genotype distribution may not represent the general population, because all enrolled individuals were from the hospital, which may lead to selection bias. Third, this study only investigated one polymorphism and did not study gene-gene interaction. Fourth, we did not detect the IL-10 levels in three kind genotypes of individuals respectively. It is of note that previous studies demonstrated that GG genotype carriers showed elevated IL-10 levels compared with other genotypes [18]. Fifth, this study did not show clinical features of AKI such as septic, ischemic, nephrotoxic, and obstructive AKI. Last, the genetic-environment interaction should be performed.

In summary, the IL-10 -1082 A/G polymorphism enhances the risk of AKI in Chinese Han subjects. Further studies in different races should be conducted to validate these findings.

## Data Availability

The datasets used and/or analyzed during the current study are available from the corresponding author on reasonable request.
